# Interaction of Avibactam with Class B Metallo-β-Lactamases

**DOI:** 10.1128/AAC.00897-16

**Published:** 2016-09-23

**Authors:** Martine I. Abboud, Christian Damblon, Jürgen Brem, Nicolas Smargiasso, Paola Mercuri, Bernard Gilbert, Anna M. Rydzik, Timothy D. W. Claridge, Christopher J. Schofield, Jean-Marie Frère

**Affiliations:** a Department of Chemistry, University of Oxford, Oxford, United Kingdom; b Laboratoire de Chimie Biologique Structurale (CBS), Département de Chimie, Université de Liège, Liège, Belgium; c Laboratory of Mass Spectrometry, GIGA-R-CART, Université de Liège, Liège, Belgium; d Centre d'Ingénierie des Protéines, Université de Liège, Liège, Belgium; e Chimie Analytique Inorganique, Département de Chimie, Université de Liège, Liège, Belgium

## Abstract

β-Lactamases are the most important mechanisms of resistance to the β-lactam antibacterials. There are two mechanistic classes of β-lactamases: the serine β-lactamases (SBLs) and the zinc-dependent metallo-β-lactamases (MBLs). Avibactam, the first clinically useful non-β-lactam β-lactamase inhibitor, is a broad-spectrum SBL inhibitor, which is used in combination with a cephalosporin antibiotic (ceftazidime). There are multiple reports on the interaction of avibactam with SBLs but few such studies with MBLs. We report biochemical and biophysical studies on the binding and reactivity of avibactam with representatives from all 3 MBL subfamilies (B1, B2, and B3). Avibactam has only limited or no activity versus MBL-mediated resistance in pathogens. Avibactam does not inhibit MBLs and binds only weakly to most of the MBLs tested; in some cases, avibactam undergoes slow hydrolysis of one of its urea N-CO bonds followed by loss of CO_2_, in a process different from that observed with the SBLs studied. The results suggest that while the evolution of MBLs that more efficiently catalyze avibactam hydrolysis should be anticipated, pursuing the development of dual-action SBL and MBL inhibitors based on the diazabicyclooctane core of avibactam may be productive.

## INTRODUCTION

**FIG 1:**
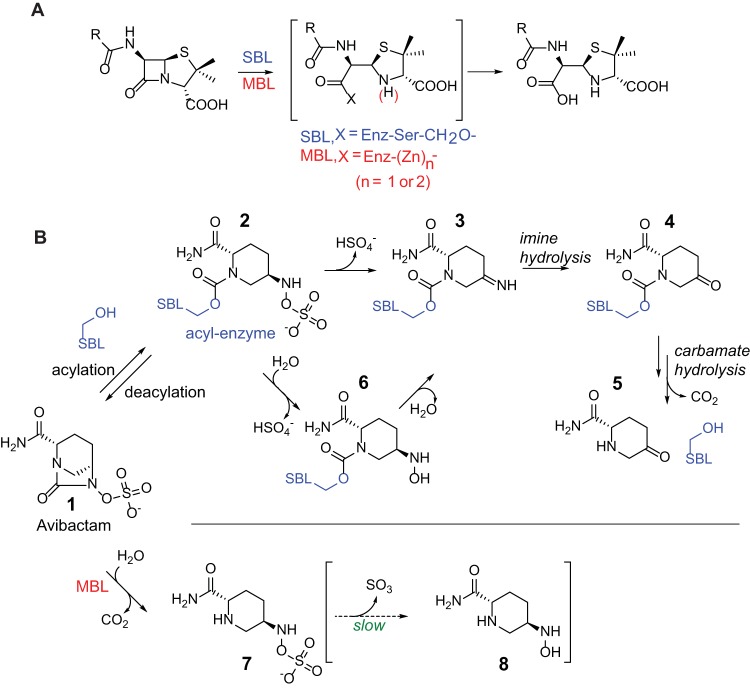
Outlines of reaction schemes for β-lactamase hydrolysis and the reactions of avibactam with SBLs and MBLs. (A) Reactions catalyzed by serine β-lactamases (SBLs) and metallo-β-lactamases (MBLs), outlining proposed intermediates. Enz, enzyme. (B) Note the variation in mechanisms for avibactam hydrolysis as catalyzed by SBLs and MBLs. With SBLs (with KPC-2), the available evidence is that hydrolysis of the acyl-enzyme complex proceeds via initial elimination to give an imine followed by acyl-enzyme ester hydrolysis (11). In the case of the MBLs, ester hydrolysis can occur without elimination and imine hydrolysis.

The β-lactams remain the most successful antibacterials; however, their effectiveness is threatened by resistance, most importantly as mediated by β-lactamases, which catalyze β-lactam hydrolysis, thus ablating antibacterial activity. In mechanistic terms, β-lactamases are classified into those enzymes that employ a nucleophilic serine residue (“serine” β-lactamases [SBLs], class A, C, and D β-lactamases) and zinc ion-dependent enzymes (the metallo-β-lactamases [MBLs] or class B β-lactamases) (1) (Fig. 1A). A major advance in β-lactam therapy was the development of class A (penicillinase) β-lactamase inhibitors (clavulanic acid, sulbactam, and tazobactam). Such compounds contain a β-lactam but have low intrinsic antibacterial activity; their utilization in combination with a penicillin antibiotic has found very widespread clinical use (1). Although this approach has been successful, resistance to the class A SBL inhibitors has emerged, and they do not usefully inhibit the class C and D β-lactamases, which catalyze hydrolysis of the important cephem and, sometimes, carbapenem classes of β-lactam antibacterials (2–4).

Avibactam (previously NXL104 or AVE1330A) has been recently (2015) approved for clinical use in combination with a cephalosporin, ceftazidime, for treatment of urinary tract and other infections caused by otherwise antibiotic-resistant bacteria (5). Aside from its use in extending the scope of cephalosporins, avibactam represents a breakthrough because unlike all of the other clinically used β-lactamase, or penicillin-binding protein (PBPs) (the targets of β-lactam antibiotics) inhibitors, avibactam does not contain a β-lactam ring. Instead, avibactam is the first of a new class of clinically useful nucleophilic serine enzyme inhibitors that contain a diazabicyclooctane (DBO) core (6, 7). Like the useful β-lactam inhibitors, avibactam efficiently reacts to form covalent adducts with SBLs, as shown by work on class A (TEM-1) and class C (P99) β-lactamases (8). However, unlike the β-lactams, which react irreversibly with the nucleophilic serine residue, in the case of avibactam, formation of the covalent adduct(s) is reversible (9), a difference likely arising substantially from the relative ease of five-membered versus four-membered ring formation (10). All class A β-lactamases that have been tested (TEM-1, CTX-M-15, and KPC-2) are potently inhibited by avibactam (11), while the class C Enterobacter cloacae P99 and Pseudomonas aeruginosa PAO1 enzymes are somewhat less sensitive. The class D OXA SBLs exhibit a wider range of sensitivities to avibactam. In the case of KPC-2, a slow, irreversible hydrolysis of the acyl-enzyme complex derived from avibactam was also observed yielding 5-oxo-piperidine-5-carboxamide after fragmentation, but this apparently does not substantially impair the efficacy of avibactam-mediated KPC-2 inhibition (11) (Fig. 1B). Various studies have reported on the ability of avibactam to restore the activity of β-lactam antibiotics against β-lactamase-producing bacteria (12–14). Avibactam restores the sensitivity of strains producing class A, C, and some D SBLs to cephalosporins (e.g., ceftazidime and ceftaroline) and the monobactam aztreonam (15–17). In contrast to the ability of avibactam to restore the activity of β-lactam antibacterials in otherwise resistant SBL-producing cells, no such effect is seen in the case of MBL-producing bacteria (note that many resistant bacteria produce both SBLs and MBLs) and avibactam does not potentiate the activity of aztreonam in MBL-producing cells (13, 18) because aztreonam is not an MBL substrate (19).

Although for several decades the MBLs were considered of little clinical relevance, they are now an increasing global threat since many MBLs (e.g., NDM, VIM, IMP, GIM, and SPM types) are plasmid encoded in their rapid dissemination and are able to catalyze the hydrolysis of almost all types of β-lactam antibiotics, with monobactams being an exception (20). Although the SBLs and MBLs catalyze the same chemical reaction, the MBLs are structurally unrelated to the SBLs. Because they do not contain a nucleophilic serine, the MBLs are not, at least beneficially, inhibited by the clinically used class A SBL inhibitors such as clavulanic acid (21).

To our knowledge, the susceptibility of avibactam to MBL-catalyzed hydrolysis has not yet been reported. This is an important issue since MBLs are becoming more widespread (22), and MBL-catalyzed avibactam hydrolysis has the potential to impair the potency of β-lactam antibiotic-avibactam combinations against strains producing both SBLs and MBLs. Here, we report that avibactam is not an MBL inhibitor and is a poor substrate for most members of all three clinically relevant subclasses of MBLs. The results suggest that while the evolution of MBLs that more effectively catalyze avibactam hydrolysis should be anticipated, the development of dual-action SBL and MBL inhibitors based on the DBO of avibactam or on other non-β-lactam cores is of interest.

## MATERIALS AND METHODS

### Abbreviations.

MBL, metallo-β-lactamase; SBL, serine β-lactamase; NDM, New Delhi MBL; VIM, Verona integron-encoded MBL; IMP, imipenemase; GIM, German imipenemase; SPM, São Paulo MBL; PBP, penicillin-binding protein; CTX-M, cefotaxime-resistant SBL from Munich; KPC, Klebsiella pneumoniae carbapenemase; BcII, Bacillus cereus MBL; P99, Enterobacter cloacae strain P99 β-lactamase; CphA, Aeromonas hydrophila AE036 carbapenemase; FEZ, Fluoribacter (Legionella) gormanii ATCC 33297^T^ lactamase; TEM, Temoniera lactamase; OXA, oxacillin-resistant carbapenemase; DBO, diazabicyclooctane; BTFA, 3-bromo-1,1,1-trifluoroacetone; NMR, nuclear magnetic resonance; DMSO, dimethyl sulfoxide; CPMG, Carr-Purcell-Meiboom-Gill; PROJECT, periodic refocusing of J evolution by coherence transfer; wLOGSY, water-ligand observed gradient spectroscopy; TFA, trifluoroacetic acid; Vis, visible; 2D, two-dimensional; HSQC, heteronuclear single quantum correlation; HMBC, heteronuclear multiple bond correlation; LC-MS, liquid chromatography-mass spectrometry; UPLC, ultraperformance liquid chromatography, CCD, charge-coupled device; NOE, nuclear Overhauser effect; COSY, correlation spectroscopy; MS, mass spectrometry; ESI, electrospray ionization.

### Chemicals and drugs.

Chemicals were from Sigma-Aldrich, unless otherwise stated. Avibactam and ceftazidime were kindly supplied by AstraZeneca and GlaxoSmithKline, respectively.

### Protein production and purification.

Recombinant forms of the Δ42-NDM-1, VIM-2, VIM-4, SPM-1, FEZ-1, CphA, IMP-1, and BcII MBLs were produced in Escherichia coli as described previously (23–27). ^19^F-labeled NDM-1 and SPM-1 (here referred to as NDM-1* and SPM-1*, respectively) were prepared by S-alkylation of cysteine variants (Cys67 in NDM-1* and Cys152 in SPM-1*) with 3-bromo-1,1,1-trifluoroacetone (BTFA) as described previously (28, 29). Recombinant Δ35-NDM-1 was a gift from David Ehmann of AstraZeneca.

### NMR binding experiments.

For FEZ-1, VIM-2, VIM-4, CphA, NDM-1, SPM-1 and BcII, NMR spectra were recorded using a Bruker AVIII 600-MHz NMR spectrometer equipped with a BB-F/^1^H Prodigy N_2_ cryoprobe using 5-mm diameter NMR tubes (Norell) or 3-mm Match NMR tubes (CortecNet). Data were processed using TopSpin 3.1 software (Bruker). For the hydrolysis of avibactam by NDM-1, ^1^H NMR spectra were acquired using a Bruker AVIII 700-MHz spectrometer equipped with a ^1^H/^13^C/^15^N TCI cryoprobe. Data were recorded with a relaxation delay of 2 s and 16 scans, employing a pulse sequence with water suppression (excitation sculpting with gradients using perfect echo) (30). The final concentration of NDM-1 was 0.022 mM. Other samples contained the MBL (80 μM) and 50 molar equivalents of avibactam (4 mM final from a DMSO stock) in Tris-D_11_ buffer (50 mM, pH 7.5) supplemented with 10% D_2_O, unless otherwise stated. More details about the NMR sequences are stated below.

### (i) ^1^H CPMG NMR experiments.

Typical experimental parameters for Carr-Purcell-Meiboom-Gill (CPMG) NMR spectroscopy were the following: total echo time, 40 ms; relaxation delay, 2 s; and number of transients, 264 (31). The PROJECT-CPMG sequence (90°*x*-[τ-180°*y*-τ-90°*y*-τ-180°*y*-τ]_*n*_-acq) was applied. Water suppression was achieved by presaturation. Prior to Fourier transformation, the data were multiplied with an exponential function with 3-Hz line broadening.

### (ii) ^1^H NMR experiments.

For ^1^H excitation sculpting suppression with perfect echo NMR experiments, spectra were typically obtained using 256 scans and a relaxation delay of 1 s (32). A 2-ms sinc pulse was used for water suppression. Prior to Fourier transformation, data were multiplied with an exponential function with 2-Hz line broadening.

### (iii) wLOGSY NMR experiments.

For water-*l*igand *o*bserved *g*radient *s*pectroscop*y* (wLOGSY) analyses, typical experimental parameters were the following: mixing time, 1 s; relaxation delay, 2 s; number of transients, 256 (33). Solvent excitation was achieved using a 16-ms 180° selective rectangular shape pulse with 1,000 points (Squa100.1000) set at the H_2_O frequency. Water suppression was achieved by a 2-ms sinc pulse (Sinc1.1000) at the H_2_O frequency.

### (iv) ^19^F NMR experiments with ^19^F-labeled NDM-1* and SPM-1*.

^19^F NMR spectra were acquired at 298 K using a Bruker AVIII 600-MHz spectrometer with a BB-F/^1^H Prodigy N_2_ cryoprobe. Data were recorded with a 2-s relaxation delay and 256 scans. For data processing, 4-Hz line broadening was used. NMR samples were prepared in 50 mM HEPES buffer (pH 7.5), 200 mM NaCl, supplemented with 10% D_2_O. Trifluoroacetic acid (TFA) (50 μM) was used as an internal standard, and its chemical shift value was set to −75.45 ppm. The final concentration of ^19^F-labeled NDM-1* (28) or SPM-1* (29) was 0.08 mM and that of avibactam was 2 mM.

### Hydrolysis of avibactam followed by UV-Vis spectroscopy.

VIM-4 (8 μg) was added to a 1 mM solution of avibactam (1 ml) in 5 mM HEPES buffer (pH 7.5) containing 35 μM ZnCl_2_. A spectrum between 210 and 280 nm was then immediately recorded. After 20 h at 20°C, another spectrum was recorded; the appearance of this spectrum was not modified after further incubation. The rate of avibactam hydrolysis was estimated by monitoring the decrease in absorbance at 230 nm (Δε = 910 M^−1^ · cm^−1^). Two buffers were used: 5 mM HEPES (pH 7.5) and 50 mM cacodylate (pH 7.0), both containing 20 μM ZnCl_2_. The MBL (8 to 75 μg, depending upon the activity) was added to a 600-μl sample of 1 mM avibactam in buffer, and the absorbance was monitored for 40 to 240 min at 30°C. Due to the low activity observed with NDM-1, incubation was continued overnight (20 h) (23).

### Inhibition of ceftazidime hydrolysis by avibactam.

Ceftazidime inhibition assays were performed in 5 mM HEPES buffer (pH 7.5) containing 20 μM ZnCl_2_. The initial rate of ceftazidime hydrolysis (30 μM) was monitored at 260 nm and 30°C in the presence or absence of 1 mM avibactam.

### Analysis of hydrolysis products. (i) NMR analyses.

NMR spectra (^1^H, 2D ^1^H-^15^N/^1^H-^13^C HSQC, and HMBC) were recorded using a Bruker Avance I 500-MHz spectrometer equipped with a TCI cryoprobe. Avibactam (1 mg/ml, 1 ml) in 10 mM sodium phosphate (pH 7.5) in D_2_O was added to 20 μg of VIM-4 enzyme.

### (ii) LC-MS analyses.

Liquid chromatography-mass spectrometry (LC-MS) analyses were performed with a UPLC system (Acquity I class; Waters) and a BEH C_18_ column (2.1 mm × 5 cm) (flow, 0.25 ml/min). Samples were loaded onto the column in 5% solvent B (acetonitrile). After 2 min, a gradient from 5% to 90% solvent B was performed over 6 min. The UPLC system was coupled to an ESI Q-Orbitrap mass spectrometer (Q Exactive; Thermo Scientific), operating either in the positive or negative ion mode.

### (iii) Raman spectroscopy.

Six hundred microliters of 1 mM avibactam in 5 mM HEPES (pH 7.5) containing 20 μM ZnCl_2_ was mixed with 12 μg of VIM-4. After 4 h at 37°C, changes in the absorbance at 230 nm indicated that ∼70% of the avibactam had been hydrolyzed. Samples (2 μl) were withdrawn after 0 and 240 min. A second 240-min sample was analyzed following addition of 2 μl of 1 M Na_2_SO_4_. The samples were deposited as solution drops and then dried on a polished stainless steel plate. They were analyzed using a LabRam 300 spectrometer (Jobin-Yvon) equipped with an Olympus confocal microscope and an Andor open electrode iDus CCD detector. The laser source used was either a Cobolt Samba 500 (532 nm) or a Melles Griot He-Ne laser (632.8 nm). The laser was focused on the target using a ×100 objective. The maximum laser beam power was limited to 1.5 mW by use of neutral density filters to minimize sample degradation. The integration times ranged from 30 to 50 s, depending on the sample. In order to check the homogeneity of the spots, spectra were recorded, with both lasers, on several areas of the sample visualized using a CCD camera. Where necessary, a baseline correction was applied to the recorded spectra using a polynomial regression model and homemade software.

## RESULTS

### Binding studies.

**FIG 2:**
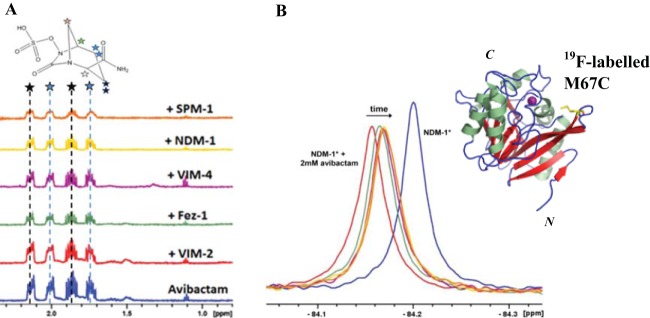
Studies of the binding of avibactam to MBLs using NMR. (A) ^1^H CPMG analyses of avibactam binding to selected MBLs imply that avibactam is a weak binder of the indicated MBLs as evidenced by a reduction of the signal intensity of avibactam when in the presence of an added MBL in solution. (B) ^19^F-NMR analysis of avibactam of NDM-1* indicates that avibactam either binds NDM-1* near its active loop or its binding induces changes in the labeled L1 loop (28). For assay conditions, see Materials and Methods.

We first used small-molecule NMR spectroscopy (ligand observed NMR methods) (see Table S1 in the supplemental material) to assay for avibactam binding to representatives of the 3 different MBL subfamilies (NDM-1, SPM-1, BcII, VIM-2, and VIM-4, B1 MBLs; CphA, B2 MBL; and FEZ-1, B3 MBL). All of these MBLs employ a dizinc active site except for CphA, which is a monozinc MBL (34). The ^1^H Carr-Purcell-Meiboom-Gill (CPMG) filtering (31) results with NDM-1, VIM-2, VIM-4, SPM-1, and FEZ-1 all indicate weak binding of avibactam (Fig. 2A); in some cases, limited avibactam hydrolysis was observed (see below). In the case of the “model” B1 MBL from Bacillus cereus BcII (35) and B2 CphA MBLs, we did not observe binding by ^1^H CPMG under our standard conditions. We did, however, accrue evidence for binding of avibactam to BcII using the sensitive water-ligand observed gradient spectroscopy (wLOGSY) (33) method, implying that avibactam binds (very) weakly to BcII (see Fig. S1 in the supplemental material). Further analyses were carried out on SPM-1 because it has structural elements of both B1 and B2 MBL subfamilies (36, 37); all of the NMR methods used (^1^H [32], CPMG [31], and wLOGSY [33]) (see Fig. S2 in the supplemental material) indicated that avibactam is a weak SPM-1 binder. While with SPM-1, wLOGSY (33) evidence for avibactam binding was accrued, no evidence for binding of products of avibactam was observed (i.e., intact avibactam and SPM-1 have the same NOE sign, while the hydrolyzed product(s) has an NOE sign opposite to that of SPM-1, characteristic of a free ligand) (see below and also Fig. S5 in the supplemental material).

For SPM-1 and NDM-1, where we observed clear evidence for avibactam binding by ligand observed NMR, we further analyzed binding using protein NMR (28, 29). SPM-1 Y152C and NDM-1 M67C variants which were ^19^F labeled using 1-bromo-3,3,3-trifluoroacetone (BTFA) were used (the S-alkylated cysteine variants are denoted SPM-1* and NDM-1*, respectively) in assays monitoring for binding by ^19^F-NMR. The ^19^F labels were positioned on a loop surrounding the active site (the α3 loop in SPM-1* [29] and the L1 loop in NDM-1* [28]), thus enabling monitoring changes in the ^19^F chemical environment on ligand binding. On addition of avibactam to either SPM-1* or NDM-1*, clear shifts and broadening of the protein-bound ^19^F signals were observed, indicating that avibactam binds to the active site region/or its binding elicits changes in the ^19^F-labeled loops. Interestingly, for NDM-1*, a time-dependent change in the chemical shift of the initially observed signal (at −84.25 ppm) was observed after avibactam treatment, with the signal moving toward, but not reaching the position observed for NDM-1* in the absence of ligand. This observation may indicate either that the reaction was incomplete or that avibactam fragments to give product(s) that bind to NDM-1* (Fig. 2B) (similar observations were noted with SPM-1*). Avibactam fragmentation is involved during its hydrolysis for some SBLs (11). Overall, the NMR results reveal that avibactam binds weakly to some but not all of the tested MBLs.

### Kinetic analyses.

For kinetic analyses, we first employed a spectroscopic assay for avibactam hydrolysis. Comparison of the UV-visible spectra indicated that the hydrolyzed product(s) of avibactam exhibits decreased absorbance relative to avibactam with the highest variation in the 220- to 230-nm region (Δε_230_ = 910 M^−1^ · cm^−1^). The rate of avibactam hydrolysis was routinely estimated by monitoring the decrease in absorbance at 230 nm in either 5 mM HEPES (pH 7.5) or 50 mM cacodylate (pH 7.0) containing 20 μM ZnCl_2_. With the FEZ-1 MBL, use of 50 mM cacodylate resulted in significant inhibition; hence, FEZ-1 experiments were repeated using a 10-fold diluted buffer. With CphA, the ZnCl_2_ concentration was reduced to 1 μM since the binding of a second Zn(II) ion is inhibitory for most B2 MBLs (38, 39). In all cases, substrate turnover did not exceed 20% in order to remain as close as possible to initial rate conditions while measuring sufficient variations in absorbance to obtain reliable results. The MBL (8 to 75 μg, depending upon the activity) was added to a 600-μl sample of 1 mM avibactam in buffer, and the absorbance was monitored for 40 to 240 min at 30°C. Due to the very low activity observed with NDM-1, incubation was continued overnight (20 h). Because of the very low reaction rates, it was not possible to directly determine the *K_m_* and *v* values. The results (Table 1) give the following order of avibactam hydrolysis efficiency in both HEPES and cacodylate buffers: VIM-4 > FEZ-1 > IMP-1 ∼ NDM-1.

**TABLE 1:** Hydrolysis of 1 mM avibactam by MBLs from different subfamilies

Enzyme	Results for HEPES buffer		Results for cacodylate buffer	
	*v*_0_/E_0_^*a*^ (min^−1^)	*k*_cat_/*K_m_* (M^−1^ · s^−1^)	*v*_0_/E_0_^*a*^ (min^−1^)	*k*_cat_/*K_m_* (M^−1^ · s^−1^)
VIM-2	1.8 ± 0.2	30 ± 3	0.7 ± 0.15	11 ± 2
VIM-4	6.0 ± 1.2	100 ± 20	3.0 ± 0.6	50 ± 10
SPM-1	1 ± 0.2	17 ± 3	0.7 ± 0.15	11 ± 2
NDM-1	ND^*b*^	<2	0.18 ± 0.04	3 ± 0.6
IMP-1	0.12 ± 0.02	2 ± 0.4 (3.5 ± 0.7)^*c*^	ND	<2
FEZ-1	4.3 ± 0.8	70 ± 15 (100 ± 20)^*c*^	2.5 ± 0.5^*d*^	42 ± 8^*d*^
CphA	0.35	6 ± 1.2	<0.1	<2

a *v*_0_/E_0_ ratios obtained for each enzyme with 1 mM avibactam are presented. Because of the very low reaction rates we did not attempt to determine the *K_m_* and *v* values directly. Approximate *K_m_* values can be calculated from the results of the inhibition experiments.

b ND, not detectable (<0.1).

c The values in parentheses are corrected as a function of the approximate *K_m_* value (see text).

d The cacodylate buffer was 5 mM.

We then carried out inhibition analyses using ceftazidime as a substrate to obtain approximate *K_m_* values. For all of the MBLs, the reported ceftazidime *K_m_* values are >30 μM (VIM-2, 72 μM [40]; SPM-1, 46 μM [36]; NDM-1, 180 μM [41]; IMP-1, 46 μM [26]; FEZ-1, >1,000 μM [42]). Since the reported conditions [buffer, Zn(II) concentration, temperature] are different from ours, we used complete time courses to verify that ceftazidime hydrolysis is quasi first-order in all cases, including for VIM-4, for which we did not find a reported *K_m_* value. Consequently, we conclude that all of the ceftazidime *K_m_* values are substantially >30 μM, so that MBL “protection” by ceftazidime is not an important factor in the analysis of the results (see Table S2 in the supplemental material).

Surprisingly, we observed some unexplained apparent activation of SPM-1-catalyzed ceftazidime hydrolysis by avibactam. For the VIM-2, VIM-4, and NDM-1 enzymes, the *K_i_* values (reflecting *K_m_* values) should be considered to be >5 mM. The limited inhibition of IMP-1 and FEZ-1 enabled us to calculate *K_i_* (= *K_m_*) values of ∼1.7 and ∼2.3 mM, respectively. These values should be considered with caution since they were derived from results obtained at a single avibactam concentration. However, the *K_m_* values were so high and the *v*_0_ values so low that it did not seem appropriate to obtain more accurate values. As a consequence, the *k*_cat_/*K_m_* values displayed in Table 1 were calculated from the *v*_0_/E_0_ values, assuming that the latter reflected a first-order process [(*v*_0_/E_0_) = (*k*_cat_/*K_m_*)|S_0_]. For the IMP-1 and FEZ-1 enzymes, a second value is also given after correction on the basis of the approximate *K_m_* value, but the corrections do not result in major differences.

### Characterization of MBL-mediated avibactam hydrolyzed product(s).

**FIG 3:**
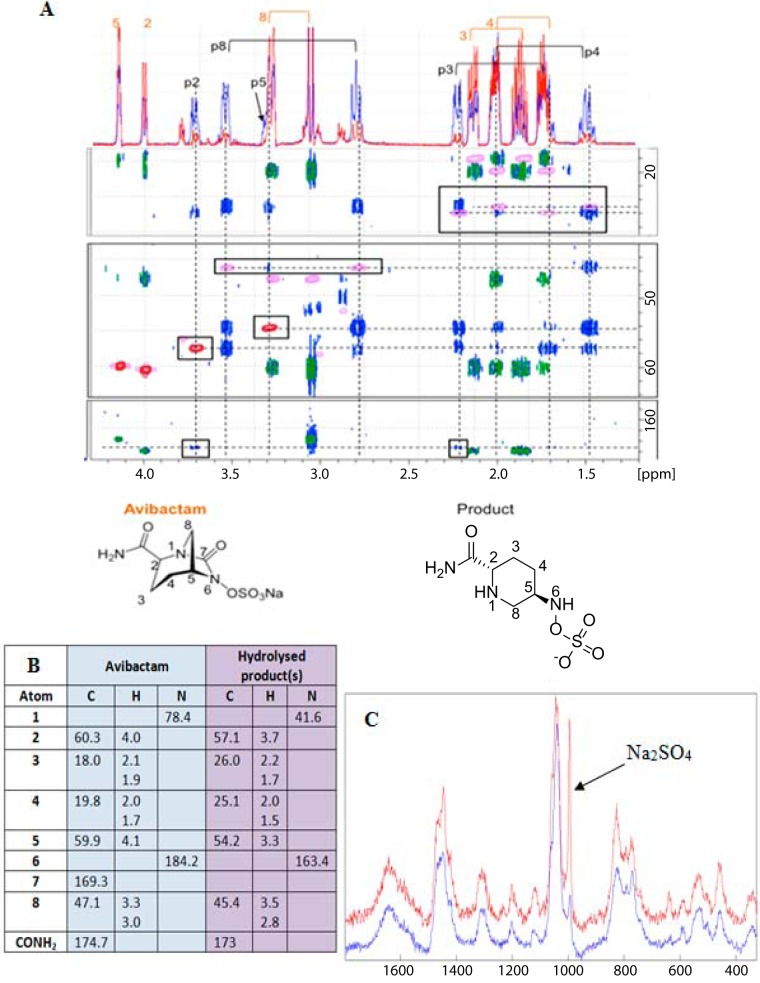
Analyses of the hydrolysis reactions of avibactam with selected MBLs by NMR and Raman spectroscopy. (A) 2D ^1^H-^13^C correlation analysis of a mixture of 50% avibactam- and 50% avibactam-hydrolyzed products. Edited HSQC: CH (red), CH_2_ (pink). HMBC: avibactam pure (green), hydrolysis-avibactam reaction mixture 50:50 (blue). Hydrolysis product signals are in the black boxes in the 2D ^1^H-^13^C correlation. The 1D ^1^H spectrum displayed at the top of the 2D plane corresponds to avibactam (red) and the hydrolysis-avibactam reaction mixture 50/50 (blue). The black labels correspond to the hydrolyzed products. Orange labels correspond to avibactam. (B) Assigned chemical shifts (parts per million) of the products of avibactam VIM-4-mediated hydrolysis are consistent with the products observed with SPM-1 and NDM-1 (2D characterization data not shown). (C) Raman spectroscopic analyses reveal that VIM-4-catalyzed hydrolysis of avibactam proceeds predominantly via simple hydrolysis (and loss of CO_2_) of the avibactam cyclic urea (Fig. 1). Blue trace, VIM-4 and avibactam after 240 min; red trace, same sample spiked with Na_2_SO_4_ (the arrow corresponds to Na_2_SO_4_). For assay conditions, see Materials and Methods.

We then assayed for MBL-catalyzed avibactam hydrolysis using small-molecule NMR. For BcII, only very low levels of hydrolysis were observed (see Fig. S3 in the supplemental material), consistent with the weak binding observed by NMR. SPM-1-catalyzed hydrolysis was also slow (see Fig. S4 and S5). For NDM-1 (see Fig. S6) and VIM-4, more efficient hydrolysis (although still extremely slow by normal β-lactamase standards) was observed to give an apparently stable product (>4 h after a 4-hour incubation for VIM-4). Controls without enzyme did not lead to detectable product levels. ^1^H NMR, COSY, HMBC, and HSQC NMR analyses implied that the products (from NDM-1, SPM-1, and VIM-4 catalysis) had lost the C7 carbonyl of avibactam but retained atoms N1 and N6 (Fig. 3A and B). The nitrogen of the CO-NH_2_ side chain was not detected due to proton exchange with the solvent. From these NMR data, it was not possible to tell with confidence whether the N-OSO_3_^−^ bond of avibactam had been cleaved during MBL-catalyzed hydrolysis (see Fig. S7).

Avibactam was observed both in positive (*m*/*z* = 266.0443, M + H^+^) and negative (*m*/*z* = 264.0295, M − H^+^) ion mass spectrometry (MS) modes. After VIM-4-catalyzed hydrolysis, the avibactam signal was significantly reduced (∼50-fold). In the negative ion mode, an intense peak at *m*/*z* 238.0499 corresponding to the hydrolyzed compound having lost a COOH group was observed ([C_6_H_13_O_5_N_3_S-H]^−^; measurement error, −1.741 ppm). This hydrolysis product was also observed in the positive ion mode (*m*/*z* 240.064; [C_6_H_13_O_5_N_3_S + H]^+^; measurement error, −1.365 ppm). An additional, less intense, signal was observed in the positive ion mode at *m*/*z* 160.108, corresponding to a loss of an SO_3_ group from the first hydrolyzed product ([C_6_H_13_O_2_N_3_ + H]^+^; measurement error, −0.8 ppm). In the positive ion mode, a very minor new compound was detected (*m*/*z* = 143.0815), corresponding to 5-oxopiperidine-2-carboxamide (C_6_H_10_N_2_O_2_ + H^+^); this product has been identified by Ehmann et al. after reaction of avibactam with the KPC-2 SBL (11). Overall, these results imply that the avibactam N-OSO_3_^−^ group is maintained in the major product of VIM-4-catalyzed avibactam hydrolysis. However, the MS assays did not enable us to unequivocally determine whether the major product observed has an N-OSO_3_^−^ group, because of the possibility that the different products show different ionization properties.

We thus used Raman spectroscopy to assay for sulfate production. Under our working conditions, free sulfate yields a sharp band just below 1,000 cm^−1^. Comparison of the Raman spectra from an incubation of avibactam with VIM-4 after 240 min with the same sample to which an identical amount of Na_2_SO_4_ had been added (Fig. 3C) reveals that the amount of sulfate produced from avibactam is very low (<15% of the total avibactam product). Thus, the major product from VIM-4-catalyzed hydrolysis contains an intact N-OSO_3_^−^ group.

Overall, these results imply that VIM-4-catalyzed hydrolysis of avibactam (and by implication) catalyzed by other MBLs proceeds predominantly via “simple” hydrolysis of the avibactam cyclic urea followed by decarboxylation rather than the more complex mechanism of hydrolysis operating for the KPC-2 serine β-lactamase (11).

## DISCUSSION

The most important outcome of the results is that, while avibactam is not an MBL inhibitor (within detection limits), at least some MBLs can catalyze the slow hydrolysis of avibactam. These results explain why avibactam does not protect β-lactams in the cases of MBL-producing pathogens. Although the MBLs tested only bind avibactam weakly and some inefficiently catalyze its hydrolysis, there is clearly an opportunity for the evolution of new MBLs/MBL variants that efficiently catalyze avibactam hydrolysis. It is therefore of interest to develop derivatives of avibactam, as new types of β-lactamase/PBP inhibitors, that are either resistant to potentially efficient MBL-catalyzed hydrolysis or are MBL inhibitors. In this regard, it is notable that the only clinically used β-lactam not known to be an MBL substrate is aztreonam (15). Like aztreonam, avibactam contains an -SO_3_^−^ group, albeit one that is directly linked to the nitrogen of the acylating heterocycle (β-lactam for aztreonam), whereas in the case of avibactam compound 1, the –SO_3_^−^ group is linked to the cyclic urea via an O atom (i.e., an RR′N-OSO_3_^−^ group). Thus, there may be an opportunity for decreasing MBL-catalyzed hydrolysis of the avibactam core by modifying its RR′N-OSO_3_^−^ group. Interestingly, avibactam protects aztreonam from hydrolysis by SBLs even in the presence of MBLs; thus, it would seem important to preserve and build on the apparent advantage of the aztreonam-avibactam combination.

A related interesting observation also relates to the RR′N-OSO_3_^−^ group of avibactam. The combined NMR, MS, and Raman studies imply that, at least in the cases we studied, MBL-catalyzed avibactam hydrolysis proceeds via simple hydrolysis of the acyl urea to give an unstable carbamate which loses CO_2_ to give a stable product, compound 7, as observed by NMR and MS (Fig. 1 and 3). By MS, we also observed some evidence for a product arising from further hydrolysis of the RNH-OSO_3_^−^ to give the RNHOH, compound 8; however, this was only a very low-level product, and we cannot be certain it results from enzyme catalysis. In contrast, with SBLs, avibactam reacts reversibly with the nucleophilic serine to give an acyl-enzyme complex, compound 2 (11, 43). The available evidence (with KPC-2) is that this complex undergoes initial β-elimination to give an imine, compound 3, prior to hydrolysis of the acyl-enzyme complex ester (Fig. 1). The imine may be hydrolyzed to the ketone, compound 4, before or after acyl-enzyme complex hydrolysis (11). Thus, pathways for β-lactamase-catalyzed avibactam hydrolysis appear to differ for the tested SBLs and MBLs.

## Supplementary Material

Supplemental material
