# Supplementary material for: Interaction of Avibactam with Class B Metallo-β-Lactamases
Source: Antimicrob Agents Chemother. 2016 Sep 23;60(10):5655–62. doi: 10.1128/AAC.00897-16 (PMC5038302; doi:10.1128/AAC.00897-16)
Supplement: Supplemental material [file supp_60_10_5655__index.html]

Interaction of Avibactam with Class B Metallo-β-Lactamases — Supplemental material 

# Interaction of Avibactam with Class B Metallo-β-Lactamases

## Supplemental material

- Supplemental file 1 -

  Fig. S1-S7 and Tables S1 and S2

  PDF, 740K
