# Supplementary material for: Interaction of Avibactam with Class B Metallo-β-Lactamases
Source: Antimicrob Agents Chemother. 2016 Sep 23;60(10):5655–62. doi: 10.1128/AAC.00897-16 (PMC5038302; doi:10.1128/AAC.00897-16)
Supplement: Supplemental material [file AAC.00897-16_zac009165510so1.pdf]

Supplementary Information

Interaction of Avibactam with Class B Metallo-β-lactamases

Martine I. Abboud,[a‡] Christian Damblon,[b‡] Jürgen Brem,[a] Nicolas Smargiasso,[c] Paola Mercuri,[d] Bernard Gilbert,[e] Anna M. Rydzik,[a] Timothy D. W. Claridge,[a] Christopher J. Schofield,[a]# and Jean-Marie Frère[d]#

Department of Chemistry, University of Oxford, Oxford, United Kingdom[a] ; Laboratoire de Chimie Biologique Structurale (CBS), Département de Chimie, Université de Liège, Belgique[b] ; Laboratory of Mass Spectrometry, GIGA-R – CART, Université de Liège, Belgique[c] ; Centre d'Ingénierie des Protéines, Université de Liège, Belgique[d] ; Chimie Analytique Inorganique, Département de Chimie, Université de Liège, Belgique[e]

Running Head: Interaction of a serine-β-lactamase inhibitor with metallo-β-lactamases

#Address correspondence to Christopher J. Schofield, christopher.schofield@chem.ox.ac.uk, and Jean-Marie Frère, jmfrere@ulg.ac.be.

‡M.I.A and C.D. contributed equally to this work.

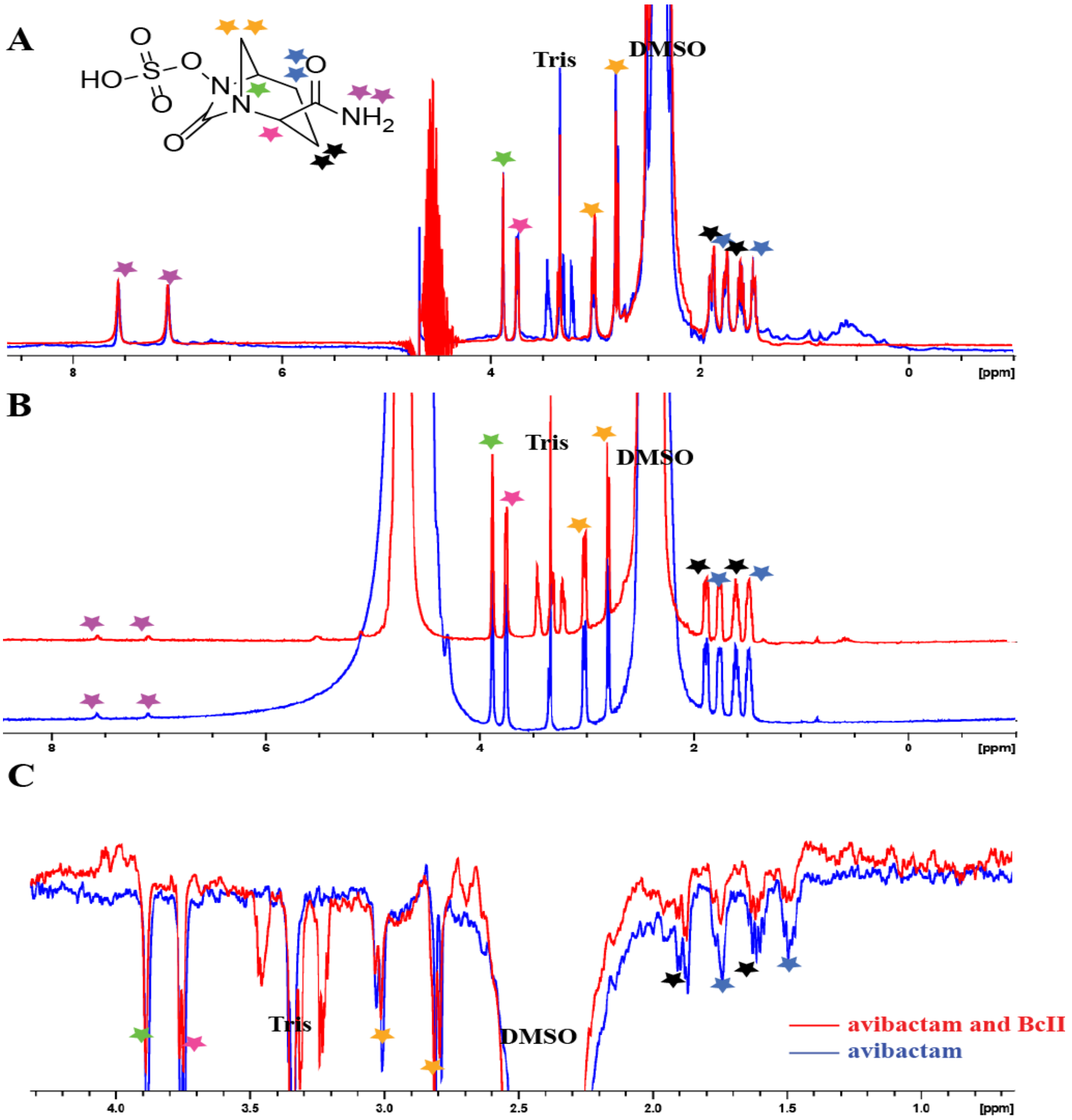

FIG S1 NMR assays for binding of avibactam to BcII. (A) ^{1}H NMR spectra of avibactam (blue trace) and BcII mixed with avibactam (red trace). (B) ^{1}H CPMG NMR spectra of avibactam (blue trace) and Bc-II mixed with avibactam (red trace). (C) wLOGSY NMR spectra of avibactam (blue trace) and Bc-II mixed with avibactam (red trace). The wLOGSY results indicate weak binding of avibactam to BcII. The assay mixtures contained 80 µM di-Zn(II)-BcII and 4 mM Avibactam in 90 % H_2O and 10 % D_2O, 50 mM Tris-D_{11}, pH 7.5.

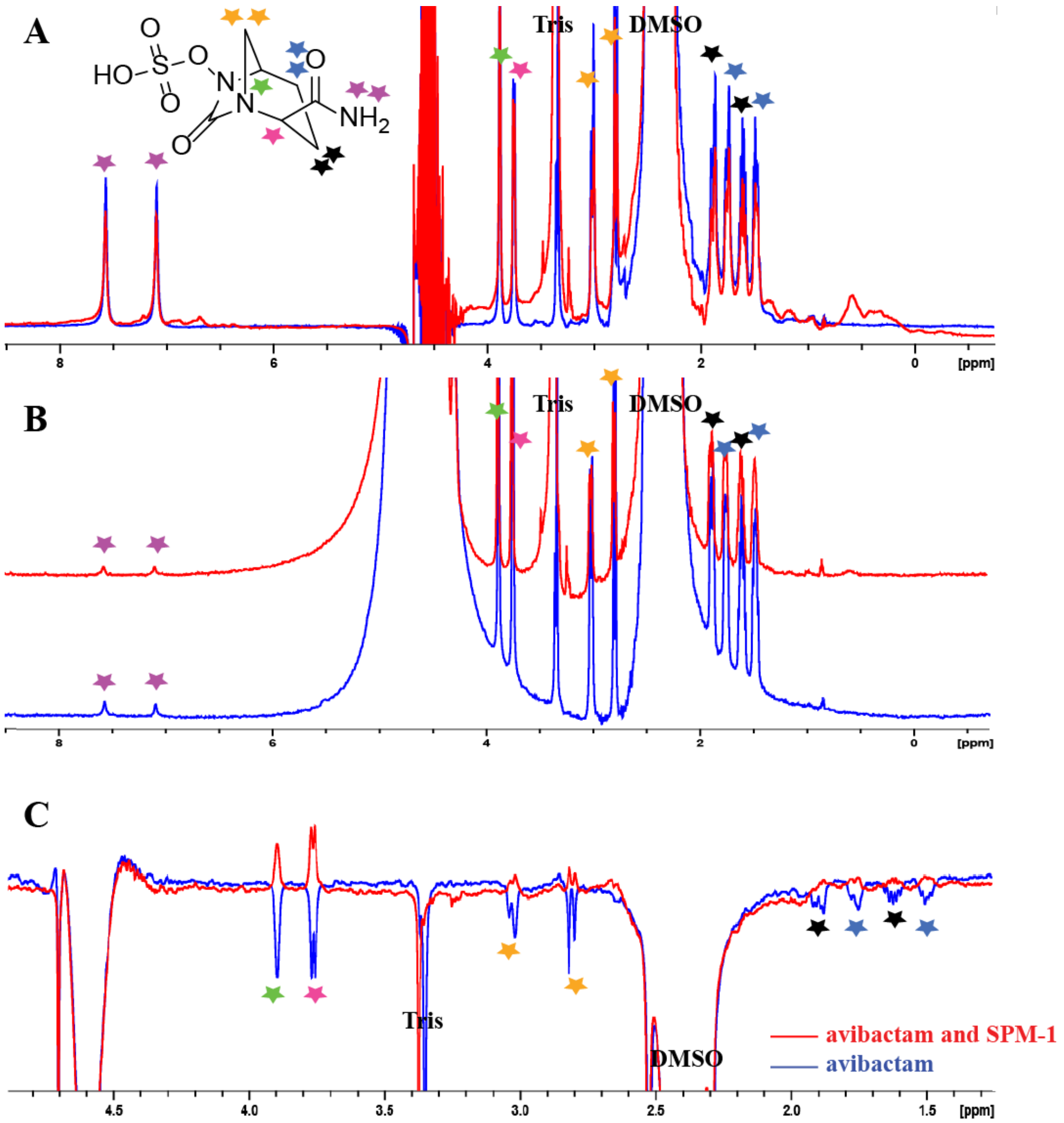

FIG S2 NMR assays for binding of avibactam to SPM-1. (A) ^{1}H NMR spectra of avibactam (blue trace) and SPM-1 mixed with avibactam (red trace). (B) ^{1}H CPMG NMR spectra of avibactam (blue trace) and SPM-1 mixed with avibactam (red trace). (C) wLOGSY NMR spectra of avibactam (blue trace) and SPM-1 mixed with avibactam (red trace). The results imply weak binding of avibactam to SPM-1. The assay mixtures contained 80 µM di-Zn(II)-SPM-1 and 4 mM Avibactam in 90 % H_2O and 10 % D_2O, 50 mM Tris-D_{11}, pH 7.5.

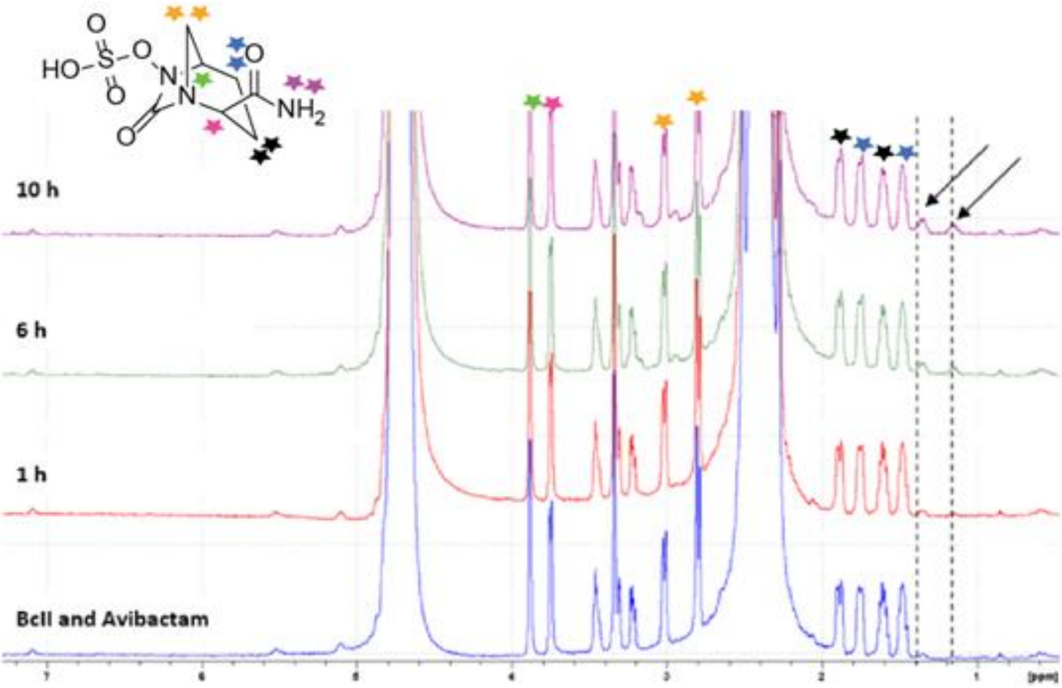

FIG S3 ^{1}H time-course CPMG NMR spectra of the reaction of BcII and avibactam monitored over 10 hours. The assay mixture contained 80 μM di-Zn(II)-BcII and 50 molar equivalents of avibactam (4 mM from a DMSO stock) in Tris-D_{11} buffer (50 mM, pH 7.5) supplemented with 10% D_2O. The appearance of peaks at 1.35 and 1.13 ppm implies that avibactam is slowly hydrolysed in the presence of BcII. Without, BcII, hydrolysis of avibactam was not observed in buffer over 10 hours. ^{1}H CPMG NMR was used to suppress signals to enable monitoring of avibactam and its hydrolyzed products.

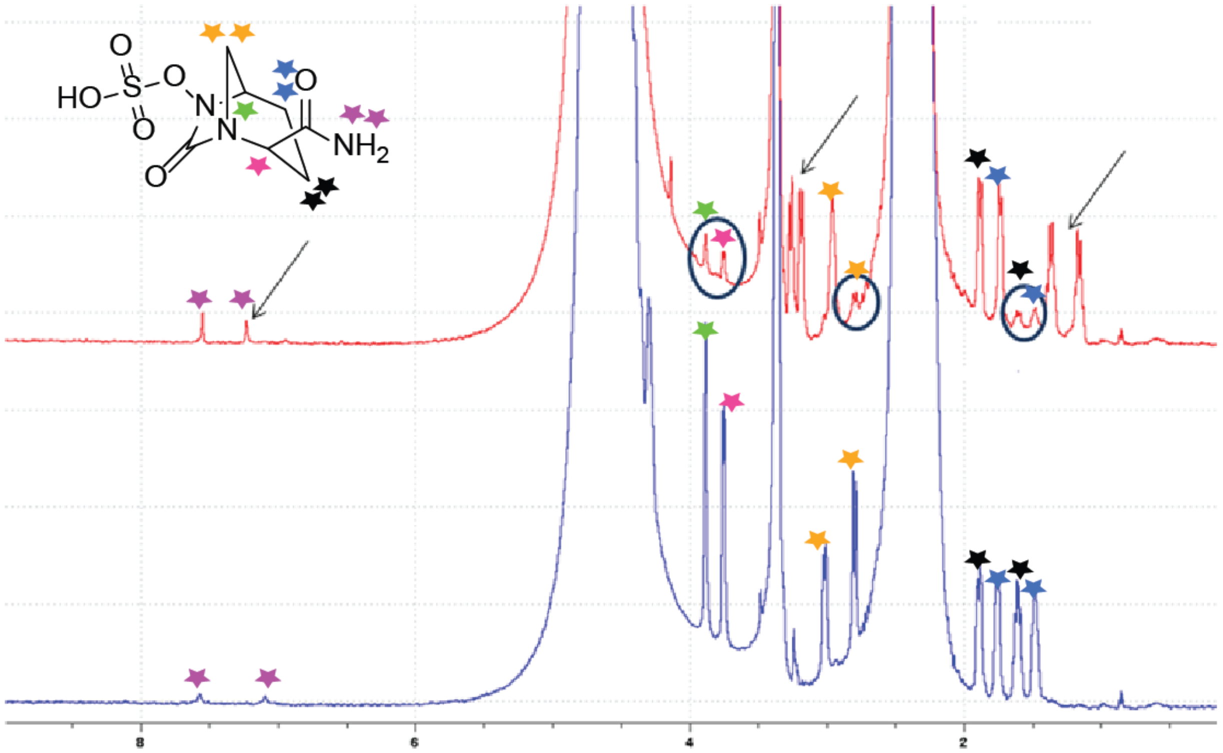

FIG S4 ^{1}H time-course CPMG NMR spectra of the reaction of SPM-1 and avibactam (upper trace, after 24 h). The assay mixture contained 80 µM di-Zn(II)-SPM-1 and 50 molar equivalents of avibactam (4 mM from a DMSO stock) in Tris-D_{11} buffer (50 mM, pH 7.5) supplemented with 10% D_2O. The data imply avibactam hydrolysis is catalyzed slowly by SPM-1. Peaks corresponding to new species are highlighted with arrows. Reductions in the major signal intensities of the intact avibactam peaks are highlighted in circles.

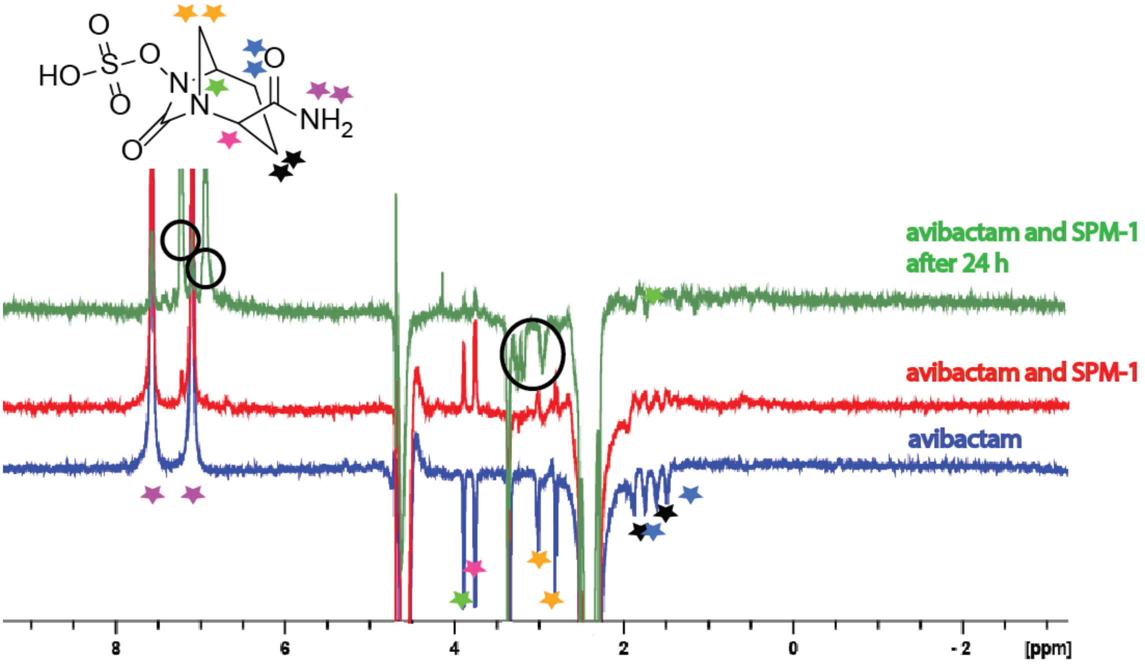

FIG S5 wLOGSY time-course analyses of the reaction of SPM-1 and avibactam (upper trace, repeated after 24h). The assay mixture contained 80 µM di-Zn(II)-SPM-1 and 50 molar equivalents of avibactam (4 mM from a DMSO stock) in Tris-D_{11} buffer (50 mM, pH 7.5) supplemented with 10% D_2O. Peaks corresponding to the new species are highlighted with arrows; we accrued no evidence for binding of this new species to SPM-1, i.e. it manifested positive NOE values as a free ligand in solution. In contrast, the results imply avibactam binds weakly to SPM-1 (**Fig. S2**).

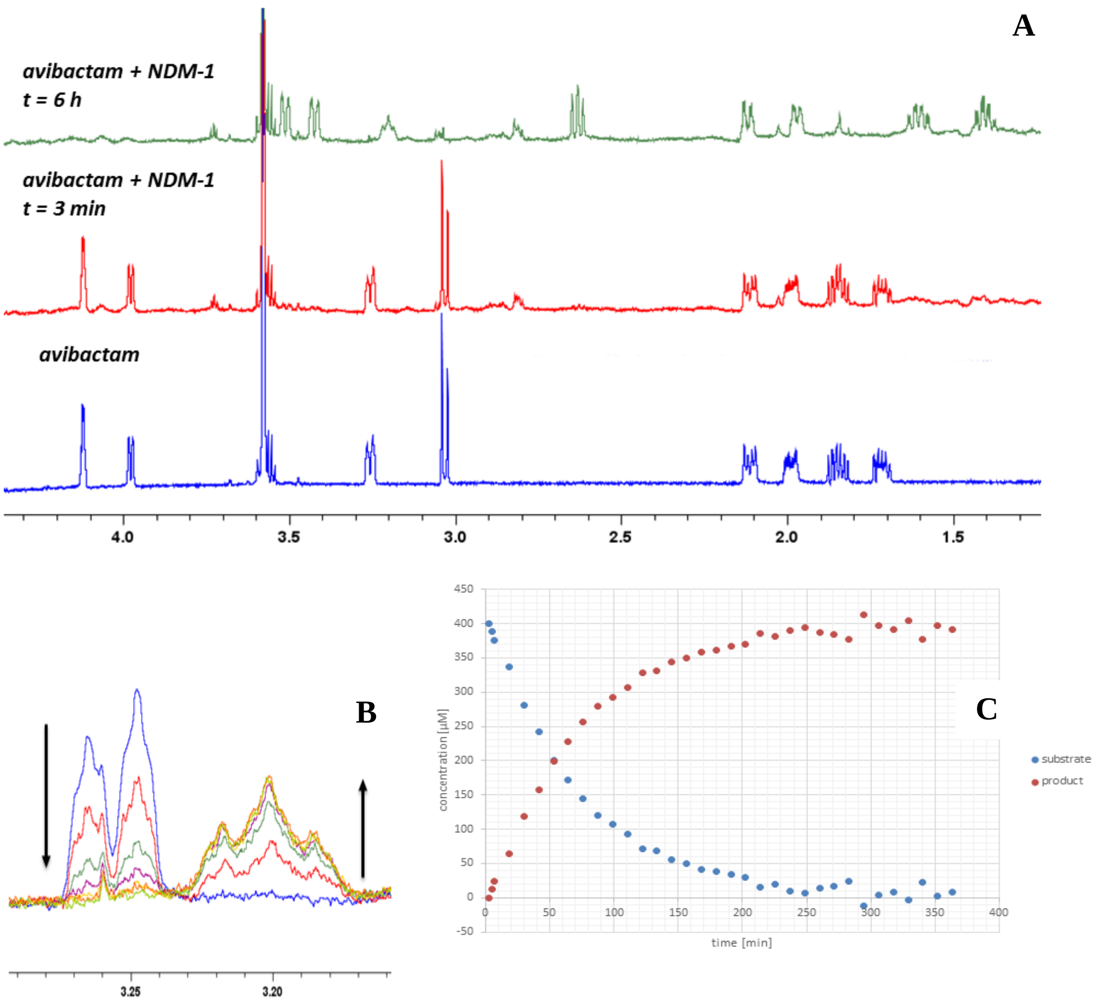

FIG S6 NMR analyses of the reaction of avibactam and NDM-1. (A) ^{1}H time-course NMR spectra of NDM-1 and avibactam. (B) Close-up view of peaks of interest. (C) Concentrations of intact avibactam and the studied hydrolysed product(s) plotted versus time. The results indicate more efficient hydrolysis of avibactam by NDM-1 compared to most other MBLs studied, except for VIM-4 (data not shown).

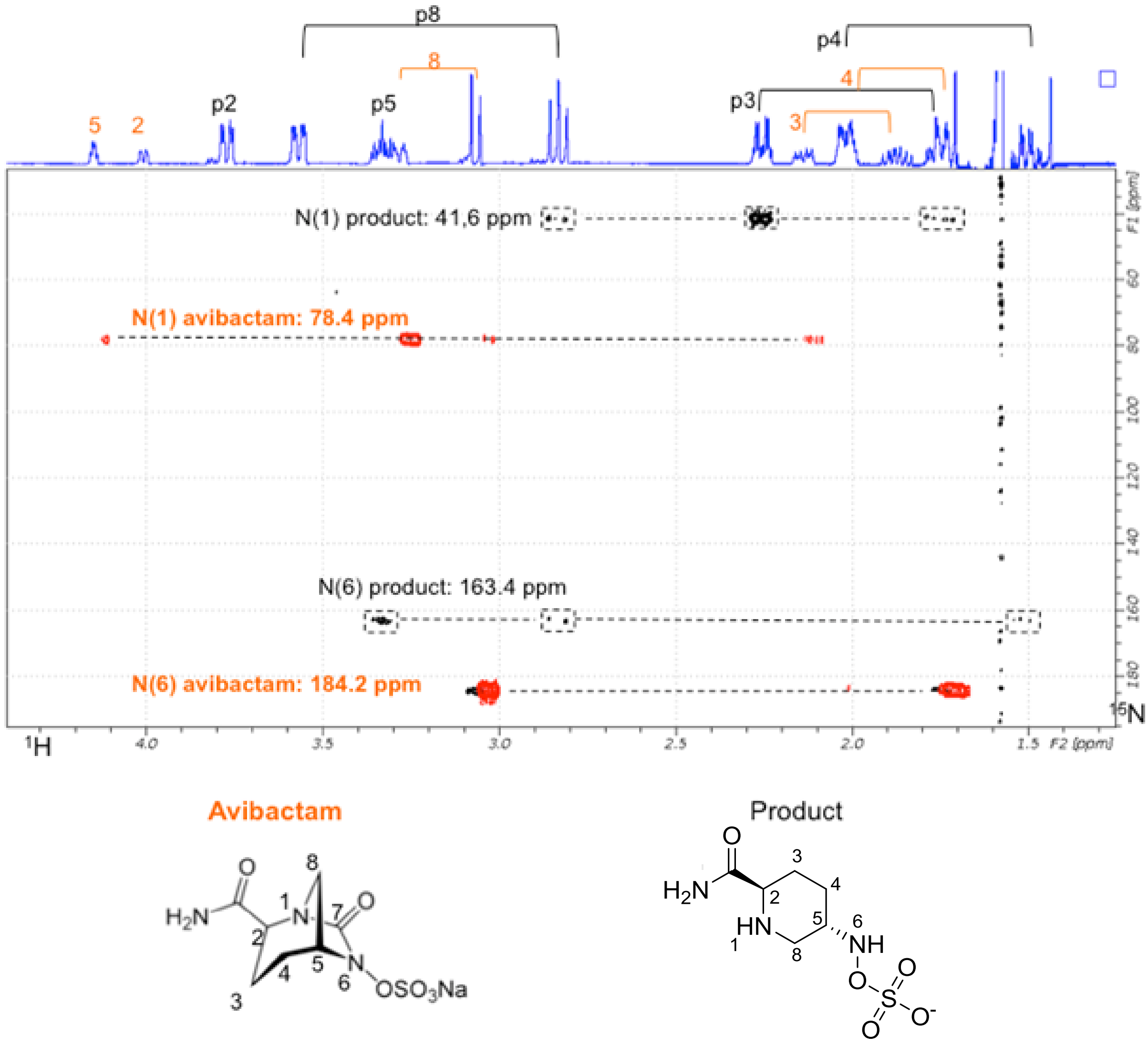

FIG S7 Characterization of VIM-4 mediated avibactam hydrolysis. The 1D ^{1}H spectrum at the top of the 2D plane corresponds to the hydrolyzed-avibactam reaction mixture 50/50 (blue). Black labels correspond to the hydrolysis product**.** 2D ^{1}H-^{15}N HMBC correlation experiment of the ~1:1 mixture of avibactam and the avibactam hydrolyzed products (black)**.** 2D ^{1}H-^{15}N HMBC correlation experiment of avibactam is in red. Orange labels correspond to avibactam.

**NMR method**	**Observations on binding *(within detection range)***
**^{1}H NMR**	line broadening
**^{1}H CPMG NMR**	intensity reduction
**wLOGSY NMR**	change of the NOE intensity in the presence of the receptor

TABLE S1 Summary of the expected observations of ligand binding to proteins as observed by ligand-observe NMR methods. For assay conditions, see Materials and Methods.

Enzyme	Residual activity (%)	Approximate K_i value (mM)
**VIM-2**	91 ± 4	>> 5
**VIM-4**	85 ± 5	>> 5
**SPM-1**	140 ± 15	? (not relevant)
**NDM-1**	90 ± 5	>> 5
**IMP-1**	60 ± 6	1.7
**FEZ-1**	71 ± 2	2.3

TABLE S2 Residual activity at ceftazidime (30 µM) in the presence of 1 mM avibactam (HEPES buffer). For assay conditions, see Materials and Methods.
